# Brief report: large individual variation in outcomes of autistic children receiving low-intensity behavioral interventions in community settings

**DOI:** 10.1186/s13034-015-0039-6

**Published:** 2015-03-25

**Authors:** Yoko Kamio, Hideyuki Haraguchi, Atsuko Miyake, Mikio Hiraiwa

**Affiliations:** Department of Child and Adolescent Mental Health, National Institute of Mental Health, National Center of Neurology and Psychiatry, 4-1-1 Ogawa-Higashi, Kodaira, Tokyo 187-8553 Japan

**Keywords:** Autism spectrum disorders, Applied behavior analysis, Early intervention, Low intensity, Outcome

## Abstract

**Background:**

Despite widespread awareness of the necessity of early intervention for children with autism spectrum disorders (ASDs), evidence is still limited, in part, due to the complex nature of ASDs. This exploratory study aimed to examine the change across time in young children with autism and their mothers, who received less intensive early interventions with and without applied behavior analysis (ABA) methods in community settings in Japan.

**Methods:**

Eighteen children with autism (mean age: 45.7 months; range: 28–64 months) received ABA-based treatment (a median of 3.5 hours per week; an interquartile range of 2–5.6 hours per week) and/or eclectic treatment-as-usual (TAU) (a median of 3.1 hours per week; an interquartile range of 2–5.6 hours per week). Children’s outcomes were the severity of autistic symptoms, cognitive functioning, internalizing and externalizing behavior after 6 months (a median of 192 days; an interquartile range of 178–206 days). In addition, maternal parenting stress at 6-month follow-up, and maternal depression at 1.5-year follow-up (a median of 512 days; an interquartile range of 358–545 days) were also examined.

**Results:**

Large individual variations were observed for a broad range of children’s and mothers’ outcomes. Neither ABA nor TAU hours per week were significantly associated with an improvement in core autistic symptoms. A significant improvement was observed only for internalizing problems, irrespective of the type, intensity or monthly cost of treatment received. Higher ABA cost per month (a median of 1,188 USD; an interquartile range of 538–1,888 USD) was associated with less improvement in language-social DQ (a median of 9; an interquartile range of −6.75-23.75).

**Conclusions:**

To determine an optimal program for each child with ASD in areas with poor ASD resources, further controlled studies are needed that assess a broad range of predictive and outcome variables focusing on both individual characteristics and treatment components.

## Background

Autism spectrum disorders (ASDs) are persistent disabling neurodevelopmental disorders that are clinically evident from early in life. Accordingly, many countries have given greater public attention to ASDs and allocated more public funds to implement and develop community services or promote research in this field. Among them, early identification and subsequent intervention for ASDs are considered key issues. A recent systematic review of early intensive intervention concluded that Lovaas-based approaches, early intensive behavioral intervention variants and the early intensive comprehensive approach (the Early Start Denver Model) resulted in some improvement in cognitive, language, and adaptive functioning in some young children with ASD compared with broadly defined eclectic treatments [1].

The growing body of evidence on early interventions for children with ASD suggests that there is great variability in children’s response to treatment [1-6]. However, the responder’s characteristics for each treatment have not been well identified, which makes it difficult for clinicians to recommend any specific form of intervention as the best option for an individual child with ASD.

In Japan, existing intervention services are generally insufficient in terms of their quantity and quality to meet the identified needs of young children with ASDs and their families. To complement existing services, various ABA-based techniques combined with parental training are provided at a limited number of universities and private agencies in metropolitan areas, although of a lower intensity. In a recent study, Hiraiwa [7] retrospectively examined the severity of autism in 60 young Japanese children with autism and found that there was a significant improvement in children receiving low intensity one-to-one treatment using various methods based on the principle of ABA (≥7 hours per week but less than the recommended intensity when compared with those receiving treatment-as-usual (TAU)); here the ABA methods included discrete trial teaching (DTT), verbal behavior (VB), and PRT provided by therapists and/or parents. Apart from case studies, Hiraiwa’s study [7] has been the only study to examine children receiving ABA in Japan. However, it used only one, less sensitive child measure, and family functioning that might have had an influence on children’s progress [8,9] was not measured.

The aim of this study was to thus explore individual outcome variations across time in young autistic children and their mothers, who received less intensive early interventions with and without ABA methods in community settings in Japan. The outcomes were assessed in terms of both child and family functioning using standardized instruments.

## Methods

### Participants

**Figure 1:**
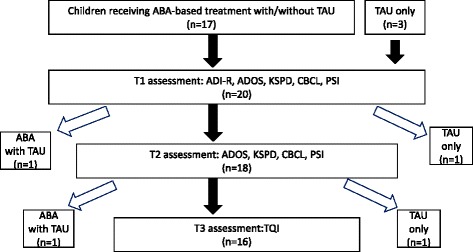
**Flowchart of the study participants.**

Seventeen children were recruited through notices posted in a specialized pediatric clinic located in a suburb of Tokyo, where one of the authors (M.H.) prescribes ABA therapy for children diagnosed with autism. In addition, three research volunteer families were contacted because they lived near the National Center of Neurology and Psychiatry (NCNP). All 20 children met the following criteria: (1) a diagnosis of autistic disorder according to DSM-IV-TR criteria corroborated by the Japanese versions of the Autism Diagnostic Interview-Revised (ADI-R) [10] and the Autism Diagnostic Observation Schedule (ADOS) [11] evaluated by an experienced child psychiatrist or psychologist with a research license; (2) an absence of medical conditions or obvious motor delay; (3) a chronological age below 7 years; (4) entry into an ABA and/or TAU program at two to five years of age. Of the 20 children, 18 (14 boys) participated in both intake and a 6-month follow-up assessment (Figure 1).

Participants’ characteristics (age, gender, scores at T1) are shown in Table 1. All participants were living with both parents. The number of siblings was similar to the national average [12]. Parental educational levels were higher than the national average [13]. Family income varied widely but the peak and mean shifted higher than those of the national average [14]. The percentage of women who were full-time housewives was higher than the national average [15].

**Table 1:** **Characteristics of the study participants**

	**Age(months)**	**Gender**	**Treatment type**	**ABA h/w T1-T2**	**ABA h/w T1-T3**	**ABA cost/m T1-T2 (USD/JPY)**	**TAU h/w T1-T2**	**TAU h/w T1-T3**	**ADI-R total score**	**ADOS CSS**	**KSPD total DQ**	**CBCL total score**	**PSI Total score**
Child 1	30	M	ABA only	4	2.8	1,000/80,000	0	0	31	5	78	59	227
Child 2	41	F	ABA only	0.5	0.7	313/25,000	0	0.3	40	10	44	58	248
Child 3	46	M	ABA only	12	14.5	5,875/470,000	0	0	32	7	85	58	185
Child 4	46	F	ABA only	4.3	4.3	1,250/100,000	0	0	28	10	67	61	237
Child 5	64	F	ABA only	2.8	2.4	5,000/400,000	0	0	23	8	82	51	142
Child 6	28	M	ABA and TAU	3	2	1,125/90,000	1	6	42	8	65	57	205
Child 7	33	M	ABA and TAU	2	2	500/40,000	3	3.5	32	8	87	63	234
Child 8	36	M	ABA and TAU	2	3.3	1,550/124,000	12.8	8.7	28	7	65	63	194
Child 9	41	M	ABA and TAU	0.3	0.3	175/14,000	0.3	0.3	33	8	90	90	265
Child 10	42	M	ABA and TAU	4	3.5	1,513/121,000	1	2.8	36	9	59	58	208
Child 11	44	M	ABA and TAU	9	7.3	3,750/300,000	14	10.5	28	7	61	66	255
Child 12	47	M	ABA and TAU	2	2.7	1,250/100,000	0.3	0.3	34	6	63	70	269
Child 13	55	M	ABA and TAU	4.3	4.3	775/62,000	0.2	0.4	35	8	74	54	160
Child 14	55	M	ABA and TAU	1	0.7	650/52,000	15.3	12.2	41	8	44	60	205
Child 15	58	M	ABA and TAU	6	6	-	4.3	4.3	41	10	37	66	177
Child 16	64	M	ABA and TAU	6.8	8.4	2,000/160,000	0.7	0.9	53	8	61	73	286
Child 17	30	F	TAU only	0	0	0	5	5	31	6	67	62	198
Child 18	62	M	TAU only	0	0	0	21.3	17.8	42	6	108	55	210
Median	45.0	-	-	3.5	3.1	1,188/95,000	0.5	0.6	33.5	8	65	60.5	209.0
IntQ	37.3-55.0	-	-	2-5.6	2-5.6	538/43,000-1,888/151,000	0-3.9	0-5.6	28.8-40.8	7-8.8	59.5-81.0	57.8-66.0	191.8-249.8
Range	28-64			0-12	0-14.5	0-5,875/0-470,000	0-21.3	0-17.8	23-53	5-10	37-108	51-90	142-286

ADOS CSS, ADOS calibrated severity score. Module 1 or 2 of the ADOS was chosen according to a child’s verbal ability. At intake, 13 children were assessed with Module 1 and five children with Module 2. At follow-up, 11 children were assessed with Module 1 and seven children with Module 2. KSPD, Kyoto Scale Psychological Development Test. IntQ = 25th percentile – 75th percentile.

The study protocol was approved by the NCNP Ethics Committee. Written informed consent was obtained from the parents of each participating child.

### Treatment

Sixteen participants received ABA-based treatments, with 11 also receiving supplemental TAU (Table 1). Five children received only ABA and two received only TAU. In addition to DTT, various ABA techniques such as VB, PRT, either alone or in combination, were provided in a one-to-one setting by highly trained therapists supervised by the program consultant. Neither therapists nor supervisors were involved in this study. Information regarding the content, hours per week, and the monthly cost of received treatment was obtained from mother-completed questionnaires at T1, T2, and T3 (Table 1). Our participants received a near average to above-average intensity of ABA as a group, and paid monthly fees to the agency/agencies that ranged from approximately US $175 to $5,875 (based on parental information, according to the currency exchange rate at the time of this study). In contrast, TAU was either free of charge or the monthly fees that were paid were less than $125. Hours of ABA/TAU per week or the cost of received treatment per month were not significantly associated with any of the child and family characteristics (child’s age, number of siblings, parental education, income). Although all parents were taught the basics of ABA and about various behavioral techniques to augment the effect of the intervention, additional ABA therapy carried out by parents themselves at home was not examined in this study.

TAU consisting of one-to-one or group programs was provided by local community-based day nurseries or specialized private preschools. The programs were organized and provided by a team that included a psychologist, nursery school teacher, community nurse, and child care staff. The frequency and hours per week of TAU provided by the community were limited across the study areas (Table 1). The TAU content was diverse with some of it including the use of picture cards or schedules, sensory integration therapy, or group-based social skills training.

### Outcome measures

Regarding child measures, although testers (licensed clinical psychologists with a master’s degree or doctoral degree) were blind to the intensity of the child’s treatment, sometimes blindness to the type of treatment was compromised unintentionally. Autistic symptoms were assessed using the Japanese version of ADOS [11]. Since the use of Calibrated Severity Scores (CSS) as an indicator of autism severity has been shown to be more valid than the ADOS raw total score [16,17], CSS were calculated from raw ADOS scores [16,18].

A child’s development was assessed using the Kyoto Scale of Psychological Development Test (KSPD) [19], which is widely used in Japanese clinical settings for young and/or developmentally delayed children and comparable to the Bayley Scales of Infant Development second edition (BSID-II) [20] (KSPD cognitive-adaptive (C-A) DQ and the BSID-II Cognitive facet, language-social (L-S) DQ and the Language facet, postural-motor (P-M) DQ and the Motor facet) [21]. Total DQs assessed by the KSPD are considered comparable to IQ scores for children with autism [22].

Children’s internalizing and externalizing behavior problems were measured using the Japanese version of the parent-rated Child Behavioral Checklist (CBCL) [23]. T-scores were used as outcome measures.

Maternal mental health was assessed using the Parenting Stress Index (PSI) and a two-question case-finding instrument (TQI). The PSI, a self-report 120-item questionnaire comprising Child and Parent domains, assesses dysfunctional parenting in parents of preschool children [24]. The TQI consisting of two questions is a depression screening tool originally included in the Primary Care Evaluation of Mental Disorders Procedure (PRIME-MD) [25]. The utility of the number of yes answers has been previously demonstrated for Japanese adults [26].

### Procedures

Eighteen participants completed both the T1 assessment (demographic information, ADI-R, ADOS, KSPD, CBCL, and PSI) and 6-month follow-up (T2) assessment (ADOS, KSPD, CBCL, and PSI). At T3 approximately 1 year after T2, the TQI and questionnaire about the received treatment were mailed to mothers, with 16 (88.9%) mothers completing and returning them (Figure 1). Time intervals T1-T2 and T2–T3 had a median of 192 days and an interquartile range of 28 days, and a median of 354 days and an interquartile range of 147 days, respectively. Performance-based tests were administered at the NCNP.

### Statistical analysis

Wilcoxon’s paired-sample test was used to compare outcome measures at T1 and T2. Since the non-normality of treatment variables was confirmed using the Shapiro-Wilk test, correlations between the predictor variables including treatment variables and child/mother measures at T1, and score changes between T1 and T2 were examined by calculating Spearman’s correlation coefficients. A Mann–Whitney test was used to compare predictor variables between participants whose mothers answered yes to one or both depression items at T3 and those whose mothers answered no to both questions. A *p*-value < .05 was considered statistically significant. The statistical analysis was performed using SPSS version 18.0 (SPSS Inc., Chicago, USA).

## Results

Table 2 provides details of each participant’s measures at T1, T2 and T3. As shown in Table 1 and Table 2, levels of children’s cognitive functioning, behavior problems and their mothers’ parenting stress at T1, treatment hours per week, treatment cost per month, and T1-T2 change in child and mother measures varied greatly in this sample. Table 3 shows the correlations between the predictor variables and T1-T2 improvement for 18 pairs of children with autism and their mothers.

**Table 2:** **Change in child and mother outcome measures**

	**Treatment type**	**ADOS CSS**		**KSPD C-A DQ**		**KSPD L-S DQ**		**KSPD P-M DQ**		**CBCL Int** ***t*** **-score**		**CBCL Ext** ***t*** **-score**		**PSI Child**		**PSI Parent**		**Mother’s depression**
		**T1**	**T2**	**T1**	**T2**	**T1**	**T2**	**T1**	**T2**	**T1**	**T2**	**T1**	**T2**	**T1**	**T2**	**T1**	**T2**	**T3**
Child 1	ABA only	5	6	83	74	78	109	-	54	63	59	46	37	99	114	128	133	0
Child 2	ABA only	10	10	46	50	25	48	60	78	65	58	49	45	127	123	121	136	2
Child 3	ABA only	7	6	69	78	112	103	44	72	63	49	51	40	99	84	86	74	0
Child 4	ABA only	10	9	67	69	73	69	43	67	67	56	52	58	119	113	118	114	1
Child 5	ABA only	8	6	85	49	80	41	71	59	49	45	44	40	67	107	75	104	0
Child 6	ABA and TAU	8	9	68	58	47	37	69	77	57	62	57	56	94	101	111	118	1
Child 7	ABA and TAU	8	7	84	82	90	84	111	89	58	57	62	61	115	115	119	132	1
Child 8	ABA and TAU	7	6	68	80	58	94	76	87	62	55	61	56	106	92	88	95	0
Child 9	ABA and TAU	8	8	85	82	93	100	90	77	85	70	65	62	140	131	125	112	0
Child 10	ABA and TAU	9	9	54	60	63	73	87	77	57	52	51	64	112	111	96	100	1
Child 11	ABA and TAU	7	7	61	57	61	67	63	73	62	52	68	54	119	102	136	106	0
Child 12	ABA and TAU	6	5	59	66	67	85	59	68	92	59	51	57	138	124	131	125	1
Child 13	ABA and TAU	8	8	69	86	83	91	-	-	54	49	53	46	91	83	69	74	0
Child 14	ABA and TAU	8	9	44	99	41	80	67	64	59	52	42	46	107	71	98	78	2
Child 15	ABA and TAU	10	9	40	55	19	42	64	-	56	54	53	51	92	82	85	75	-
Child 16	ABA and TAU	8	8	56	61	67	78	57	-	67	63	63	68	136	140	150	143	0
Child 17	TAU only	6	7	67	65	61	47	69	66	70	70	49	53	108	119	90	104	-
Child 18	TAU only	6	6	120	115	97	109	71	-	59	56	53	54	101	101	109	109	0
Dif T2-T1																		
	Median	0		3		9^✝^		9.5		−5.00***		−1.50		5		−2		
	IntQ	−.25-1.00		−4.25-9.75		−6.75-23.75		−10.25-18.50		−10.25--2.75		−5.50-4.25		−4.75-14.00		−8.50-10.50		
	Range	−2-2		−36-55		−39-39		−22-28		−33-5		−14-13		−36-40		−30-29		0-2

***p = .001, ^✝^ *p* = .08.

KSPD, Kyoto Scale Psychological Development Test. C-A, cognitive-adaptive; L-S, language-social; P-M, postural-motor. Int, internalizing; Ext, externalizing; IntQ = 25th percentile – 75th percentile.

**Table 3:** **Correlation between Predictor Variables and T1-T2 Behavior Improvement**

	**Improvement in children’s behaviors T1-T2**							**Reduction in mothers’ parenting stress T1-T2**	
	**ADOS CSS**	**Total DQ**	**C-A DQ**	**L-S DQ**	**P-M DQ**	**CBCL Int**	**CBCL Ext**	**PSI Child**	**PSI Parent**
ABA hours per week T1-T2	.27	.20	.19	−.12	.50**	−.15	−.15	.21	.39
TAU hours per week T1-T2	−.30	.12	.13	.25	−.13	.31	−.28	.27	.25
ABA + TAU hours per week T1-T2	−.07	.32	.30	.30	.31	.13	−.14	.41	.43 #
ABA cost per month T1-T2	.47^✝^	−.37	−.31	−.55**	.21	−.11	−.41	−.22	.06
Chronological age at T1	.36	.32	.29	.14	.41	−.16	.15	.18	.34
ADOS CSS at T1	.21	−.01	.25	−.02	.20	−.00	.25	.07	−.03
Total KSPD DQ at T1	.18	−.44	−.48**	−.46*	−.30	.05	−.24	−.33	−.19
CBCL total at T1	.09	.17	.19	.24	−.00	−.30	.18	.36	.50*
PSI total at T1	−.15	−.08	−.20	.17	−.20	−.36	.31	.07	.32

**p < .05, *p = .053, ^✝^ *p* = .064, ^#^ *p* = .075.

ADOS CSS, ADOS calibrated severity score. KSPD, Kyoto Scale Psychological Development Test. C-A, cognitive-adaptive; L-S, language-social; P-M, postural-motor. CBCL Int, internalizing; Ext, externalizing.

### T1–T2

Overall, a significant improvement was observed for internalizing *t*-scores (*p* = .001) only. The L-S DQ changes approached near significance (*p* = .08) (Table 2).

### Change in children’s behaviors

As shown in Table 3, ABA hours per week were significantly correlated with an improvement in P-M DQ only (*p* = .036). TAU hours per week were not associated with any change. The monthly fee paid for ABA was significantly negatively correlated with an improvement in L-S DQ (*p* = .027), although it was positively correlated with an improvement in ADOS CSS, which approached statistical significance (*p* = .064). The improvement in the child measures listed in Table 3 were not significantly associated with clinical characteristics assessed at T1, although DQs at T1 were negatively correlated with the changes in C-A and L-S DQs (*p* = .043, .053, respectively). The changes in each child measure, ADOS CSS, KSPD DQ, and CBCL, were not significantly correlated with each other, whereas among the KSPD DQs the changes in C-A DQ were correlated with those in L-S DQ and P-M DQ (*r*_*s*_ = .51, .45, *p* = .031, .064, respectively).

### Change in mother’s parenting stress

Neither ABA nor TAU hours per week were significantly associated with an improvement in the PSI Child or Parent domain scores. ABA plus TAU hours per week were associated with a reduction in PSI Parent scores, which approached statistical significance (*p* = .075) (Table 3). The monthly cost of ABA was not significantly correlated with change in either PSI Child or PSI Parent domain scores. A reduction in the PSI Child domain scores was significantly correlated with an improvement in children’s C-A DQ (*r*_*s*_ = .67, *p =* .002) and CBCL internalizing scores (*r*_*s*_ = .69, *p* = .001), while a reduction in the PSI Parent domain scores was significantly correlated with children’s CBCL internalizing scores (*r*_*s*_ = .52, *p* = .026).

### T3

#### Mothers’ depression items at T3

The frequency distribution of TQI positive items (*n* = 16) was similar to that in a recent Japanese adult sample [26]. Participants whose mothers answered yes to either one or both depression items (n = 9) did not significantly differ in either ABA or TAU hours per week, the monthly ABA cost between T1 and T3 (not shown), or family characteristics when compared with the other children (n = 7), but had a significantly increased ADOS CSS (*p* = .046) and a lower total DQ (*p* = .071) at T1.

## Discussion

We prospectively monitored the developmental progress of 18 children diagnosed as having autistic disorder who received various combinations of ABA (median 3.5 hours per week, range 0–12 hours per week) and/or TAU (median 0.5 hours per week, range 0–21.3 hours per week), and assessed their autistic symptoms, cognitive functioning, internalizing and externalizing problems at intake and 6-month follow-up, and their mothers’ mental health at intake, 6-month follow-up, and 1.5-year follow-up. Large individual variations in outcomes were observed in this study, which is consistent with the findings from previous research [2,6,27]. A significant improvement at the group level was observed only for internalizing problems, irrespective of the type and intensity of received treatments or ABA cost per month. Changes in children’s autistic symptoms, cognitive or language functioning and mothers’ parenting stress were not associated with either ABA or TAU hours per week at an individual level.

However, it was impossible to tease out the effect of ABA from that of TAU in this study. Recent studies have reported that young children with ASDs who received 2 years of lower intensity one-to-one behavioral treatment (4–15 hours per week, where the average hours per week were much higher than those in this study) showed significant progress in a broad range of parameters compared to children who had received TAU treatment [28,29]. The question of whether the intensity or duration of low intensity ABA-based treatment of the type delivered in this study is associated with an improvement in child and family functioning should be examined in future prospective controlled studies.

Regarding the outcome predictors in children, initial IQ has been identified as a strong predictor in 4 of the 11 studies of early intensive behavioral interventions [2] and in a naturalistic study [6]: that was not the case in this study (although our findings may have been affected by the small sample size). As ASD treatment predictors as well as goals can vary according to the socio-cultural context (similar to general mental health issues [30]), future intervention studies should include more diverse race/ethnic/cultural factors to better understand their effects [31].

This study has a number of methodological limitations. First, the sample size was small. Second, we obtained information on ABA and TAU treatment only through parents. We therefore lacked information on its specific form or quality. The fidelity of ABA programs delivered was not monitored. And we did not systematically evaluate parental involvement in home-based ABA therapy. Third, this study did not have a control group receiving a different type of treatment. Fourth, the blindness of the assessment was not perfect. The strengths of this study include a uniform assessment protocol with well-standardized measures of child diagnostic and developmental status as well as parental mental health [1,2].

As emphasized by Howlin et al. [2], treatment should not demand extensive sacrifice in terms of time, money, or any other aspect of family life, instead it should benefit all involved. Although our preliminary results should be interpreted with caution, they suggest that in countries such as Japan with poor ASD resources, we need to focus on individual characteristics and to think about what components should comprise an optimal program for the child with autism.

## Abbreviations

ABA: Applied behavior analysis
TAU: Treatment-as-usual
DQ: Developmental quotient
TQI: Two-question case-finding instrument
